# Characterization of Stratum Corneum Molecular Dynamics by Natural-Abundance ^13^C Solid-State NMR

**DOI:** 10.1371/journal.pone.0061889

**Published:** 2013-04-23

**Authors:** Sebastian Björklund, Agnieszka Nowacka, Joke A. Bouwstra, Emma Sparr, Daniel Topgaard

**Affiliations:** 1 Division of Physical Chemistry, Center for Chemistry and Chemical Engineering, Lund University, Lund, Sweden; 2 Leiden/Amsterdam Center for Drug Research, Department of Drug Delivery Technology, University of Leiden, Leiden, The Netherlands; National Research Council of Italy, Italy

## Abstract

Despite the enormous potential for pharmaceutical applications, there is still a lack of understanding of the molecular details that can contribute to increased permeability of the stratum corneum (SC). To investigate the influence of hydration and heating on the SC, we record the natural-abundance ^13^C signal of SC using polarization transfer solid-state NMR methods. Resonance lines from all major SC components are assigned. Comparison of the signal intensities obtained with the INEPT and CP pulse sequences gives information on the molecular dynamics of SC components. The majority of the lipids are rigid at 32°C, and those lipids co-exist with a small pool of mobile lipids. The ratio between mobile and rigid lipids increases with hydration. An abrupt change of keratin filament dynamics occurs at RH = 80–85%, from completely rigid to a structure with rigid backbone and mobile protruding terminals. Heating has a strong effect on the lipid mobility, but only a weak influence on the keratin filaments. The results provide novel molecular insight into how the SC constituents are affected by hydration and heating, and improve the understanding of enhanced SC permeability, which is associated with elevated temperatures and SC hydration.

## Introduction

Humans can be seen as water-rich bodies in a dry environment. Still, we do not desiccate. This fact is thanks to the barrier function of the skin, which upholds homeostasis and prevents the entrance of exogenous chemicals into the body. The barrier function is assured by the outermost layer of the skin, the stratum corneum (SC) [1]. Even though the SC barrier is crucial for homeostasis, there are situations where diffusional transport across the SC is desired, for instance in transdermal drug delivery applications [2]. Optimization of such processes requires fundamental understanding of the molecular organization of the SC and how it can be controlled by external factors.

The thin SC membrane (ca. 20 µm in human skin) is composed of anucleated epidermal cells (corneocytes) embedded in an extracellular multilamellar lipid matrix [3]. The SC lipids comprise a mixture of mainly cholesterol, fatty acids, and ceramides [4]. The corneocytes are filled with keratin filaments, which are enclosed by the cornified envelope (CE). The CE consists of cross-linked proteins [5] and a lipid monolayer covalently bound to the proteins [6]. It is striking that the major fraction of both the protein material inside the corneocytes and the extracellular lipids form solid structures [7], [8]. A small fraction of the extracellular SC lipids is in a mobile disordered state [7], [8], [9], and the properties of these lipid domains are still largely unexplored. In relation to the barrier properties of the SC, it is clear that the high fraction of solid SC components can assure low permeability. However, in a composite membrane with fluid and solid domains, diffusional transport preferentially occurs in the fluid regions due to the higher permeability. A molecular description of diffusional transport over the composite SC membrane therefore requires characterization of the fluid and solid SC lipid and protein components, and how these are distributed in the complex brick-and-mortar structure that builds up the SC [10]. It is important to also include the proteins in this description, as these are the major components of SC. Finally, the proportion of solid and fluid lipid and protein components can be altered by changes in the membrane environment, which can lead to dramatic changes in skin barrier properties. This coupling has previously been treated in a self-consistent theoretical model for diffusional transport in membranes composed of stacked lipid bilayers [11], [12].

In the present study, we investigate the effects of hydration and temperature on the dynamics of the molecular components in intact pig SC using natural-abundance ^13^C solid-state NMR. The experimental approach used here has recently been shown to yield atomically resolved qualitative information on molecular dynamics in surfactant [13], [14], lipid [15], amyloid fibril [16], and cartilage [17] systems. Magic-angle spinning (MAS) combined with high-power ^1^H decoupling [18] provides ^13^C NMR spectra of sufficient resolution to differentiate between the molecular moieties of the SC components, while the signal intensities observed with the CP (cross polarization) [19] and INEPT (insensitive nuclei enhanced by polarization transfer) [20] pulse sequences give information on the rate and anisotropy of molecular segment reorientation. CP and INEPT are traditionally applied to solids and isotropic liquids, respectively, in order to enhance the ^13^C signal in comparison to what is obtained with the ^13^C direct polarization (DP). We have used the acronym PT ssNMR (polarization transfer solid-state NMR) for our way of displaying overlaid and color-coded DP, CP, and INEPT spectra from which information on dynamics can be obtained by simple visual inspection [13].

Despite the complexity of the crowded ^13^C NMR spectrum from SC, we are able to perform a detailed ^13^C peak assignment using reference experiments on SC model lipids and SC from which the lipids have been extracted (i.e., isolated corneocytes). The PT ssNMR observables permit an estimation of the dynamic state of the SC components as a function of hydration and temperature. In particular, PT ssNMR is sensitive to mobile molecular segments, while it simultaneously gives information on the rigid segments in the very same sample. With this information in our hands, we have a tool to gain completely novel molecular insight into the mobility of SC lipids and proteins and how they are affected by the external conditions. Of particular significance is the new information on the fluid SC components (proteins and lipids), which are difficult to characterize with diffraction methods and until now largely uncharacterized. We observe that the majority of the SC components are completely rigid, except for a small fraction of mobile lipids, at dry conditions at 32°C. Upon hydration, there is an increasing fraction of mobile lipids and protruding terminals of the keratin filaments, while the backbone of the filaments remains almost completely rigid under all conditions explored. There is an abrupt change in dynamics of the keratin filaments at RH = 80–85%, with a transition between solid filaments and a structure with rigid backbone and hydrated mobile protruding terminals. Heating has a strong effect on the dynamics of mobile lipids, but only minor influence on the protein structures.

We show that PT ssNMR is a powerful technique for the characterization of SC and provides new molecular details of the effects of water and heat on the dynamics of the SC components. These new details provide important novel information to complement previous studies of structural alterations of the skin barrier under similar conditions [8], [21], [22], [23]. With the combined information on SC structure and molecular dynamics, together with previous theoretical and experimental studies of diffusional transport in responding membranes [11], [24], we can now provide a molecular explanation for the increased permeability of the SC upon hydration and heating [25], [26], [27], [28].

## Materials and Methods

### Materials

Synthetic ceramides (CER) were generously provided by Cosmoferm (Delft, The Netherlands). Cholesterol, free fatty acids, trypsin, chloroform, and methanol were obtained from Sigma-Aldrich. NaCl, Na_2_HPO_4_⋅2H_2_O, KH_2_PO_4_, KNO_3_, K_2_SO_4_, and KCl were purchased from Merck. Phosphate buffered saline, PBS (130.9 mM NaCl, 5.1 mM Na_2_HPO_4_⋅2H_2_O, 1.5 mM KH_2_PO_4_, pH 7.4), was prepared from Milli-Q water.

### Preparation of Stratum Corneum (SC)

Pig ears were obtained with permission from a local abattoir (Strömbecks, Brösarp, Sweden). Skin tissue from the inside of the outer ear was dermatomed and placed on filter paper soaked in PBS, containing 0.2 wt% trypsin, at 4°C for 12 h. Sheets of SC were removed with forceps and rubbed with cotton tipped applicators to remove tissue not belonging to SC, and further washed in PBS solution. In order to minimize the time required for equilibration at different RH, the SC sheets were pulverized into a flaky powder with the use of a mortar and pestel after being dried in a vacuum desiccator for two days. Comparison of NMR spectra of SC sheets and pulverized SC showed no differences and comparison of replicate SC samples showed good reproducibility. NaCl solutions of different concentrations (5.12, 4.02, 2.81, 2.02, 1.19, 0.156 mol kg^−1^) were used to regulate the RH (80.0, 85.0, 90.0, 93.0, 96.0, 99.5% RH, respectively) at 32°C. SC was weighed dry before and after 48 h of equilibration time. The water content is defined as (*m*_SC,humid_-*m*_SC,dry_)/*m*_SC,humid_ where *m*_SC,dry_ is the dry weight and *m*_SC,humid_ is the mass of the humidified SC. Precaution was taken in respect to bacterial growth by treating SC with NaN_3_ (0.2 wt% in PBS) before drying. No signs of bacterial growth could be identified by comparing NMR spectra of SC with or without NaN_3_ pretreatment, which indicates that the SC samples remain stable throughout the equilibration step. The preparation without NaN_3_ was therefore used. The samples were transferred to 4 mm solid-state NMR rotors (Bruker, Karlsruhe, Germany) with inserts of approx. 15 μl volume.

### Lipid Extraction, Isolation of Corneocytes, and Preparation of SC Model Lipids

Pulverized SC was placed in 60 ml chloroform:methanol (2 h in each of the following compositions 2∶1, 1∶1, 1∶2). The SC material was collected each time by filtration. This sequence was repeated one more time with 30 min as extraction time. The SC material was then extracted overnight in methanol. Finally the SC material (containing the isolated corneocytes) was rinsed in water and dried. The extracted lipid fraction was put in a rotary evaporator to remove the organic solvents, and finally dried in vacuum. The extracted lipid sample was hydrated at 99.5% RH at 32°C for 48 h before transferred to a solid-state NMR rotor. A DP ^13^C NMR spectrum of the extracted lipid sample is presented in Figure S1 (*D*). Due to signs of potential contamination or degradation products in the extracted lipid sample (see Figure S1) we used a SC model lipid sample with known composition for peak assignment. The SC model lipid sample comprised an equimolar mixture of synthetic ceramides, cholesterol, and free fatty acids (1∶1:1). The synthetic CER mixture consisted of 15, 51, 16, 4, 9, 5 mol % of CER EOS (C30), CER NS (C24), CER NP (C24), CER AS (C24), CER NP (FA C16), CER AP (C24). The free fatty acid fraction was a mixture of C16, C18, C20, C22, C23, C24 and C26 in a molar ratio of 1.8∶4.0∶7.7∶42.6∶5.2∶34.7∶4.1. The total weight of the lipid sample was 20 mg and the mixture was prepared with a Linomat IV (Camag, Muttenz, Switzerland). The spraying rate was 5 μL/min and the solvent was evaporated by a stream of nitrogen gas on a mica substrate in an area of 10x10 mm. The silicon substrate with the applied lipid film was then equilibrated twice for 10 min at a temperature of approximately 70°C. After each heating step the sample was slowly cooled down to room temperature in approx. 30 min. After hydration in a buffer solution for 16 hours at 37°C, the sample was transferred to a solid-state NMR rotor insert under argon.

### Solid-state NMR

NMR experiments were performed on a Bruker Avance-II 500 spectrometer (Karlsruhe, Germany), equipped with a 4 mm CP/MAS HX probe, at ^1^H and ^13^C resonance frequencies of 500 and 125 MHz, respectively. ^13^C spectra were acquired at a magnetic field of 11.74 T under 68 kHz TPPM ^1^H decoupling [29], using a spectral width of 250 ppm and an acquisition time of 50 ms. The recycle delay was 5 s and 2048 scans were collected for each PT ssNMR pulse sequence, giving a total experimental time of 9 h per sample. The frequency of the magic-angle spinning (MAS) was set to *ω*_R_/2*π* = 5 kHz. The ^13^C spectra were externally referenced to the methylene signal of solid α-glycine at 43.7 ppm [30]. CP experiments were performed with the contact time *t*_CP_ = 1 ms, ^13^C nutation frequency *ω*_1_^C^/2*π* = 80 kHz, and ^1^H nutation frequency *ω*_1_^H^/2*π* linearly ramped from 72 to 88 kHz. The delays *τ* = 1.8 ms and *τ* ´ = 1.2 ms were used in INEPT. The temperature was varied using a BVT-2000 temperature control and cooling of the bearing air by a BCU-05 unit. The target temperature was chosen to obtain the sample temperatures 32, 42, and 60°C, taking into account sample heating induced by MAS and radiofrequency pulses [31]. The experimental time-domain data was processed with line broadening of 10 Hz, zero-filling from 1597 to 8192 time-domain points, Fourier transformation, automatic phase correction [32], and baseline correction in MATLAB (www.mathworks.com) using in-house code partially derived from matNMR [33].

### Theory of Polarization Transfer Solid-state NMR (PT ssNMR)

The SC membrane is a highly complex biomembrane, comprising a wide range of different molecular species with different dynamical properties. In this section we will give a brief theoretical background of the PT ssNMR technique and how it provides molecular resolution, even for complex samples such as the SC, and, more importantly, enables one to probe changes of the mobility of the resolved molecular segments.

The natural-abundance ^13^C solid-state NMR measurements employ magic-angle spinning (MAS) with the refocused INEPT [20], [34] and ramped-CP [19], [35] polarization transfer schemes for selective signal enhancement of molecular segments in either mobile (INEPT) or rigid (CP) microenvironments. The selective signal enhancement is related to how the magnetization is transferred from ^1^H nuclei to neighboring ^13^C. In the INEPT scheme the polarization transfer occurs via through-bond scalar couplings (*J*_CH_), which are unaffected by bond reorientation. INEPT yields signal enhancement as long as the ^1^H and ^13^C transverse relaxation times (*T*_2_^H/C^) are longer than the time required for ^1^H-^13^C polarization transfer, which is typically a few milliseconds. For rigid molecular segments with slow and/or anisotropic reorientations the non-averaged ^1^H-^1^H and ^1^H-^13^C dipolar interactions leads to fast *T*_2_^H/C^ relaxation and inefficient INEPT polarization transfer. For mobile molecular segments with isotropic reorientation the time-averaged dipolar interaction vanishes, thus removing the main mechanism for signal decay in the INEPT signal. In the CP scheme the magnetization is transferred via through-space dipolar couplings and the rate of this process is determined by the cross polarization time constant *T*_CH_. As pointed out, the dipolar interactions are averaged to zero for molecular segments undergoing fast and isotropic reorientations. Thus, CP enhancement is only efficient for rigid molecular segments with slow and/or anisotropic reorientations. The efficiency of CP is also dependent on the ^1^H longitudinal relaxation time in the rotating frame (*T*_1ρ_^H^), which in some situations is too fast for CP to be effective.

We have recently carried out a more in-depth theoretical analysis of the relationship between the enhancement efficiency of INEPT and CP signals and the anisotropy and dynamics of a ^13^C-^1^H_2_ segment under MAS conditions [14]. Molecular motion in heterogeneous systems is generally anisotropic and occurs on different time scales, e.g. <ps for bond vibrations, ps-ns for bond reorientations of side-chains in peptides or trans-gauche isomerization in lipid acyl chains, ns-ms for molecular rotations, and ms-s for protein folding or molecular exchange of lipids between differently oriented domains. All these types of motion can affect the reorientation of the C-H bond vector and therefore also the local magnetic field fluctuations. For a ^13^C nucleus in a ^13^C-^1^H_2_ segment, the local magnetic field is mainly determined by the directly bonded protons and is, for simplicity, described by a two-step correlation function by using the following three parameters: |*S*_CH_|, *τ*_c_, *τ*_s_ [14]. The C-H bond order parameter |*S*_CH_| and the reorientational correlation time *τ*_c_ quantifies the anisotropy and the rate of C-H motion, respectively, while the slow correlation time *τ*_s_ describes the global motion, such as molecular exchange between anisotropic domains [14]. The spectral density is obtained from the Fourier transform of the correlation function and used to calculate the relevant relaxation and polarization parameters (*T*_2_^H/C^, *T*_1ρ_^H^, and *T*_CH_), which in turn are used as input parameters to model the polarization transfer intensities [14].

**Figure 1:**
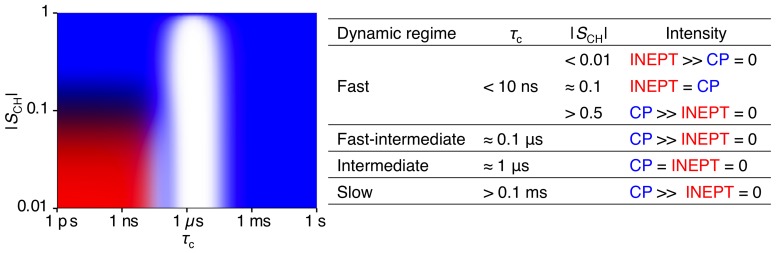
Dynamic regimes and resulting signal intensities from PT ssNMR experiments. Theoretical ^1^H to ^13^C polarization transfer efficiency as a function of correlation time *τ*_c_ and order parameter |*S*_CH_| for a CH_2_ segment at the magnetic field 11.74 T and the magic-angle spinning frequency 5 kHz, calculated with input parameters equal to the present experimental settings (see *Solid-state NMR*). The map is color-coded according to the calculated intensities of the INEPT (red) and CP (blue) polarization transfer schemes. White represents inefficient polarization transfer for both INEPT and CP. Typical values of *τ*_c_ and *S*_CH_, in the different dynamic regimes, and the expected intensities for the INEPT and CP polarization transfer schemes, are listed to the right of the figure. Adopted from ref. [14].

Under the present experimental settings the model gives the ^1^H to ^13^C polarization transfer efficiencies summarized in Fig. 1 for a CH_2_ molecular segment at varying *τ*_c_ and |*S*_CH_|. Analogous calculations for CH or CH_3_ segments yield nearly identical results. Fig. 1 also includes a compilation of typical values of *τ*_c_ and *S*_CH_, and the expected intensities for the INEPT and CP polarization transfer schemes in the different dynamic regimes. In brief, the range of *τ*_c_ can be divided into the following regimes with regards to the ^13^C signal intensities obtained with the INEPT and CP schemes: fast (<10 ns), fast-intermediate (≈ 0.1 µs), intermediate (≈ 1 µs), and slow (>0.1 ms). In the fast regime, the CP and INEPT intensities are independent of *τ*_c_ but vary with |*S*_CH_|. For nearly isotropic reorientation, |*S*_CH_| <0.01, the CP signal is unobservable while INEPT reaches maximum efficiency. Equal CP and INEPT signals are obtained when |*S*_CH_| ≈ 0.1. At high anisotropy, |*S*_CH_| >0.5, the CP signal is maximized while the INEPT amplitude is zero. In the transition between the fast and fast-intermediate regimes, the INEPT signal is gradually decreasing to be replaced by moderately efficient CP. Both CP and INEPT are inefficient in the intermediate regime, while the CP signal is maximized in the slow regime.

In particular, the model takes into account the effect of rotor spinning rate *ω*_R_ and CP contact time *t*_CP_ on *T*_2_^H/C^, *T*_1ρ_^H^, and *T*_CH_ [14]. The experimental parameters (i.e. *ω*_R_ and *t*_CP_) affect the dynamic regimes where INEPT and CP signal enhancement are efficient, and are therefore chosen to yield defined regimes where either the INEPT or the CP signal is favored. For example, increasing *ω*_R_ enhances the INEPT signal by reducing the static interaction and, thus, the transverse ^1^H relaxation rate. In addition, an extension of *t*_CP_ may favor the CP signal by polarization contributions from ^1^H-^1^H spin diffusion from distances larger than one C-H bond as long as the *T*_1ρ_^H^ is not too fast. Considering the latter aspect, the experimental setting of *t*_CP_ is chosen so that the ^1^H-^1^H spin diffusion, which is not treated in the model, can be assumed to be negligible, under which conditions the CP signal enhancement mainly reflects local polarization transfer (i.e. within approx. one C-H bond length).

For the purpose of this paper, the presence of INEPT signal is chosen as a convenient operational definition of the term “mobile”. Interpreting an increasing amplitude of the INEPT signal in terms of “increasing mobility” is unfortunately ambiguous; the reason could be one or a combination of the following: 1) a solid-to-liquid phase transition, 2) faster dynamics in the transition between the fast-intermediate and fast regimes, or 3) decreasing anisotropy in the fast regime. Prior knowledge about the studied material is often sufficient to distinguish between the different cases. Still, this caveat should be kept in mind when interpreting CP and INEPT data. In ambiguous cases, making a comparison with the DP amplitude could be fruitful. With apporiate precautions when choosing experimental parameters, the DP amplitude is expected to be fairly quantitative in the dynamical regimes where INEPT yields signal [14].

## Results and Discussion

The influence of water and temperature on the mobility of SC molecular components was investigated in a series of SC samples with varying water content at 32, 40, and 60°C by PT ssNMR measurements. We first present a detailed peak assignment of most molecular segments of the SC protein and lipid compartments. Then, we focus on major “signature peaks”, representing both protein and lipid components, and demonstrate the effects of water and temperature on the molecular dynamics of the SC components. Finally, the results are considered in relation to previously observed changes of the SC permeability in response to changes in hydration or heating.

### Peak Assignment of the ^13^C NMR Spectrum of Intact SC

**Figure 2:**
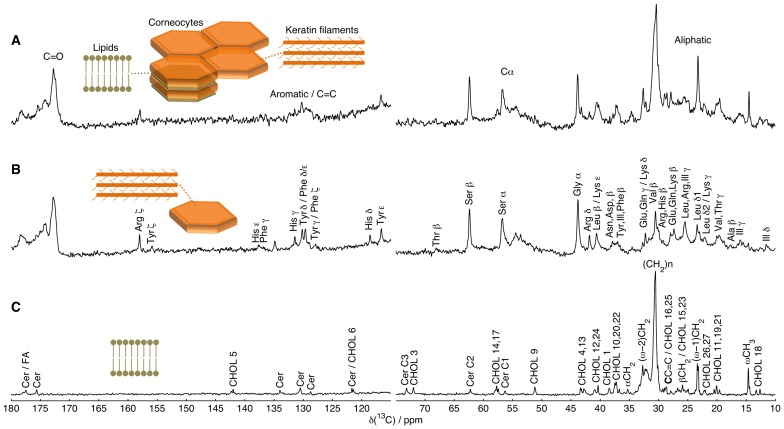
Peak assignment of molecular segments in the SC. ^13^C DP MAS NMR spectra of (*A*) intact SC, (*B*) isolated corneocytes, and (*C*) SC model lipids at 60°C. The schematics illustrate SC with corneocytes filled with keratin filaments, surrounded by a multilamellar lipid matrix. Peaks originating from the keratin and the lipids are assigned in (*B)* and (*C)*, respectively, following the IUPAC nomenclature. For peaks assigned to several amino acid residues, the names are ordered according to the expected abundance. Spectra *A* and *B* are scaled to equal intensity at 172.8 ppm, while spectra *A* and *C* are scaled to give equal intensity at 30.4–30.6 ppm. See Figure S1 (*E*) for standard numbering of cholesterol carbons and labels of lipid carbons.

The peak assignment of intact pig SC is based on DP spectra of the corneocyte sample, SC model lipid sample, and extracted SC lipids. To confirm the assignment of some molecular segments, INEPT experiments with variable delay times [34] were used, which can distinguish between CH_3_, CH_2_, and CH segments. A schematic representation of the SC is shown in Fig. 2 together with ^13^C NMR spectra from (*A*) intact SC, (*B*) isolated corneocytes, and (*C*) SC model lipids chosen to facilitate the peak assignment. From a chemistry viewpoint, the SC is a complex mixture of molecular species, which is reflected in the multitude of ^13^C resonance lines that can be observed in the spectrum from intact SC in Fig. 2 *A*. The spectrum of the isolated corneocytes in Fig. 2 *B* does not contain any peaks originating from the extracellular lipid lamellae matrix, thus displaying peaks mainly from amino acid residues. These ^13^C resonances were assigned using literature data for solubilized keratin filaments [36] and keratin filaments in the solid-state [37]. In the aliphatic region, around 30 ppm, several amino acid residues yield signals at approx. the same chemical shifts, which in some cases results in a non-resolved cluster of peaks. The spectrum of the SC model lipids in Fig. 2 *C* was assigned using literature data for fatty acids [38], cholesterol [39], and sphingosine [40], see Figure S1 (*E*) for cholesterol and relevant lipid carbon labels.

An obvious complication is that many peaks in Fig. 2 *A* (intact SC) are observed in both Fig. 2 *B* (SC proteins) and in Fig. 2 *C* (SC model lipids), which in many cases makes it difficult to exclusively assign a peak to a specific molecular segment. However, by recognizing the relative occurrence of molecular segments in SC it is possible to identify relevant markers of either the protein or the lipid matrix. Considering that dry SC consists of roughly 85 wt% proteins and 15 wt% lipids [41] it is not surprising that the majority of the peaks still remain after lipid extraction as seen by comparing spectra *A* and *B* in Fig. 2. The interior of the corneocyte comprises foremost keratin filaments and some associated protein material such as filaggrin. The principal keratin filaments in the SC are K1 and K10 [42], which are enriched in glycine and serine. About 24% of the total number residues in the amino acid sequence of K1 and K10 is glycine, while the corresponding percentage for serine is around 14% (UniProt ID P04264 and P13645). The cornified envelope is also enriched in glycine (approx. 50 wt%) and serine (approx. 20 wt%) [42]. Thus, glycine and serine, in particular, are the most abundant amino acid residues of the protein material and this is confirmed in Fig. 2 *A* and *B* where the prominent peaks from Gly C_α_, Ser C_α_, and Ser C_β_ are located at 43.7, 56.7, and 62.4 ppm, respectively. Around 90% of the glycine and 75% of the serine residues are located in the N- and C-terminal domains of the keratin filaments, thus making the Gly C_α_, Ser C_α_, and Ser C_β_ peaks suitable for probing the terminal domains (UniProt ID P04264 and P13645). Leucine and lysine are highly enriched in the coiled-coil core of the keratin filaments with about 90% and 80%, respectively, of the total number of these residues located here (UniProt ID P04264 and P13645). Consequently, the peak at 40.6 ppm from Leu C_β_ and Lys C_ε_ can be used to probe the keratin filament core. Neither of the Gly C_α_, Ser C_α_, Ser C_β_, Leu C_β_, Lys C_ε_ peaks suffer from overlap with the major peaks from the SC model lipids in Fig. 2 *C*, and are thus good markers for the protein structures of the corneocytes.

The carbons of the lipids represent a minor fraction of the total number of carbons of the SC. However, most of the lipid species contain very long saturated hydrocarbon chains in the range of C14–C32, the most common in pig SC being lengths of C20 (sphingosine-derived chain), C24 (acyl chain) for ceramides [43], and C22/C24 for fatty acids [41]. The majority of the lipid chain carbons resonate within a narrow chemical shift range from 30 to 34 ppm as seen in Fig. 2 *C*. The value of the chemical shift of a methylene group in a long hydrocarbon chain is determined by the fractions of trans and gauche conformations; for instance, the methylene resonance of a crystalline all-trans chain is about 34 ppm, while the chemical shift of methylene groups in chains having a liquid-like distribution of trans/gauche conformations is approx. 31 ppm [44]. The methylene peak around 30.5 ppm is dominant for both the intact SC and the SC model lipid samples in Fig. 2 *A* and *C*, but significantly reduced after lipid extraction in Fig. 2 *B*. A similar decrease in intensity upon extraction is also observed for the terminal methyl/methylene carbons of the lipid chains (*ω*CH_3_, (*ω*−1)CH_2_, and (*ω*−2)CH_2_ at 14.6, 23.3 and 32.7 ppm, respectively). These molecular segments are only represented by one carbon in each individual lipid chain, and are therefore not as prominent as the main methylene peak (CH_2_)*_n_*. It should be noted that these lipid peaks are reduced, but still existing to some extent after lipid extraction, cf. Fig. 2 *B*, the reason being that the corneocytes have tightly bound lipid envelopes constituting near 2 wt% of the total dry SC mass [6]. This lipid envelope is not removed with the non-hydrolytic extraction used here. Cholesterol is also a major component of the lipid matrix, but the majority of the 27 carbons of cholesterol are chemically inequivalent and their resonances are spread out over different chemical shifts. Consequently, the peaks ascribed to cholesterol in Fig. 2 *C* are buried under the protein peaks in Fig. 2 *A*. Taken together, the (CH_2_)*_n_* peaks around 30.5 and 34 ppm, along with the *ω*CH_3_ peak at 14.6 ppm and the (*ω*−1)CH_2_ peak at 23.3 ppm, represent good markers for the lipid matrix. A complete peak assignment is compiled in Table S1.

### Water Affects the Molecular Mobility of the SC Components

**Figure 3:**
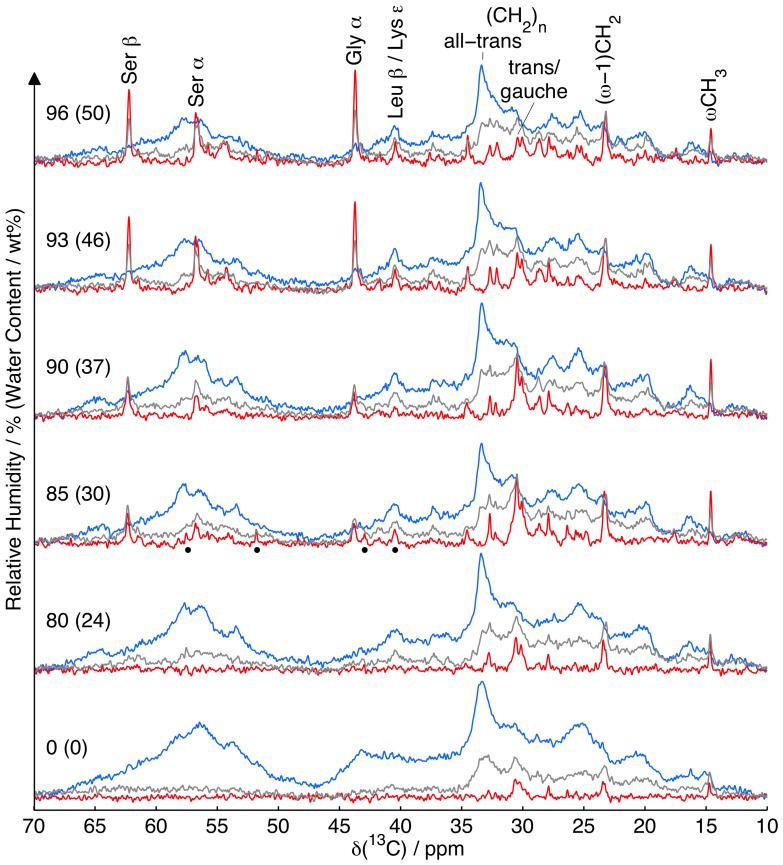
Water affects the molecular mobility of the SC components. ^13^C MAS NMR spectra of intact SC as a function of RH (water content) at 32°C using DP (grey), CP (blue), and INEPT (red) pulse sequences for preferential enhancement of the signals from molecular segments in either rigid (CP) or mobile (INEPT) microenvironments. Prominent resonance lines from keratin (Leu C_β_, Lys C_ε_, Gly C_α_, Ser C_α_, Ser C_β_) and lipids (all-trans and trans/gauche (CH_2_)_n_, (*ω*−1)CH_2_, *ω*CH_3_) are labeled in the data obtained at 50 wt% water. The dots in the 30 wt% water spectra indicate peaks of cholesterol (C12/24, C4, C14/17 and C9, cf., Fig. 2 *C*).

The effect of hydration on the molecular mobility in intact pig SC at 32°C is shown in Fig. 3. Based on the detailed ^13^C assignment in Fig. 2, a smaller number of peaks were selected as “signature peaks” for the lipids as well as the keratin core and protruding terminals as indicated in Fig. 3. Although only a small subset of the resonance lines are mentioned in the following discussion, we want to emphasize that essentially all of them are assigned, as detailed in Table S1. Qualitative information on molecular mobility is obtained by comparing the efficiency of INEPT and CP signal enhancement as a function of water content.

#### Effect of hydration on the SC lipids

The majority of the constituents of the SC are rigid, as indicated by the dominance of the CP signal for most of the spectral range. Considering the SC chemical composition, the keratin filaments give the main contribution to the CP signals. One notable exception is the comparatively sharp CP peak at 33.4 ppm, which is assigned to rigid lipids with an all-trans conformation of the methylene groups in the central part of the hydrocarbon chains [44]. The 33.4 ppm peak is practically unaffected by SC hydration, implying that the majority of the lipids remain in a rigid all-trans conformation throughout the range of studied water contents. This observation is in line with X-ray diffraction studies on SC showing only minor changes of the repeat distance of the lamellar structures [8], [23].

One important observation from Fig. 3 is that a small fraction of mobile lipids is existing in the intact SC even at completely dry conditions as inferred from the low-amplitude INEPT peaks at 14.6, 23.3, and 30.5 ppm, assigned to the *ω*CH_3_, (*ω*−1)CH_2,_ and (CH_2_)*_n_* segments of the hydrocarbon chains, respectively. This finding is in agreement with our previous ^1^H NMR study of extracted SC lipids, where a fraction of the lipids was found to be mobile even in the dry state [9]. With increasing hydration, there is a general trend of increasing amplitude of the lipid INEPT and DP peaks indicating a larger fraction of fluid lipids. Previous electron spin resonace (ESR) measurements on SC using partitioning fatty acid spin probes also suggest increased hydrocarbon chain fluidity upon hydration [45]. Similarly, IR spectroscopy studies on SC showed that hydration leads to a small, but significant, increase in the lipid-associated C-H symmetric stretching frequency, implying an increased fractional population of gauche confomers in the lipid hydrocarbon chains [21].

The relative intensities of the INEPT peaks vary with the water content; for instance, the peak intensities decrease in the order (CH_2_)*_n_*>*ω*CH_3_ at RH = 85% (30 wt% water) and *ω*CH_3_> (CH_2_)*_n_* at RH = 96% (50 wt% water). At RH = 85%, INEPT signals assigned to mobile cholesterol are visible at 40.4–40.9 (C24/C12), 42.8 (C4), 51.8 (C9), and 57.6 (C14/C17) ppm as indicated by dots in Fig. 3. The presence of these resonance lines coincides with the maximum amplitude of the lipid (CH_2_)*_n_* INEPT signal, and they are not visible at neither higher nor lower hydration. The relative decrease of the (CH_2_)*_n_* peak in comparison to the (*ω*−1)CH_2_ and *ω*CH_3_ peaks upon hydration, as well as the limited range at which cholesterol INEPT peaks are visible, is probably caused by a changing chemical composition of the fluid lipid phase, which affects the rate of molecular reorientation and consequently the INEPT efficiency. As stated above, the hydrocarbon chain lengths of the SC lipids vary in the range from C14 to C32, with the majority being C22, C24 or C26 [41], [43]. Preferential melting of short-chain species would give additional weight to the peaks from *ω*CH_3_ and (*ω*−1)CH_2_ at 14.6 and 23.3 ppm. For a hydrocarbon chain anchored to a hydrophobic-hydrophilic interface, there is a trend of decreasing values of the rotational correlation time *τ*_c_ from the headgroup towards the end of the tail [46]. The gradient in *τ*_c_ could give rise to a situation where the *ω* and (*ω*−1) carbons are in the fast dynamical regime with respect to polarization transfer efficiency, while the carbons further away from the chain end have *τ*_c_ above tens of nanoseconds and INEPT intensity close to zero. From a molecular dynamics point of view, these lipids are “fluid” although some parts of the molecules have too slow dynamics to be “mobile” according to operational definition based on INEPT efficiency. We have recently observed such behavior for the glass-forming surfactant *n*-octyl-*β*-D-maltoside [14]. An increasing fraction of long-chain species, which reorient more slowly than their short-chain counterparts, would cause a decreasing INEPT efficiency for the molecular segments contributing to the (CH_2_)*_n_* peak at 30.5 ppm. The reorienation of the C-H bonds in the steroid ring structure is determined by the whole-body motion of the entire cholesterol molecule, which is orders of magnitude slower than, e.g., trans-gauche isomerization at the end of the hydrocarbon chains. The cholesterol dynamics is sufficiently fast to give rise to INEPT signal only in the narrow hydration window at RH = 85%. We suggest that hydration induces a monotonically increasing amount of fluid lipid phase, the dynamics of which is gradually slowing down on account of the changing chemical composition: from short-chain fatty acids at low hydration to increasing fractions of long-chain fatty acids, ceramides, and cholesterol at higher hydration. These larger molecules have an inherently slower rate of reorientation than the smaller ones observed at low hydration, and they also cause a slowing down of the smaller molecules by increasing the viscosity of the fluid lipid phase.

INEPT and CP resonances with identical line shapes and comparable amplitudes are generally observed for anisotropic liquid crystalline phases of surfactants [13], [14] and lipids [15]. None of the lipid INEPT peaks in Fig. 3 are accompanied by the CP peaks expected for, e.g., a lamellar liquid crystalline phase. From this observation we conclude that the C-H bonds reorient with |*S*_CH_| <0.01, corresponding to the fluid lipids being located in a nearly isotropic microenvironment, which is in agreement with our previous ^1^H NMR study of extracted SC lipids [9]. Both anisotropic and isotropic fluid phases have been detected in model mixtures of SC lipids using ^2^H NMR of isotopically labeled free fatty acids or ceramides [47], [48]. Although the isotropic phase is universally observed at high temperatures, above approx. 70–80°C, a composition with bovine brain ceramide type III extract (mainly C18∶0, C24∶1 and C24∶0), cholesterol, and behenic acid (C22∶0), featured a coexistence between a solid and an isotropic fluid phase even at moderate temperatures, 25–50°C [47]. This result for a synthetic mixture of SC model lipids in a bulk sample is remarkably in line with our new data for intact SC, where lipids at their natural composition are confined in the narrow gaps between corneocytes. It should, however, be noted that the fraction of unsaturated ceramides is considerably higher in bovine brain than in the human SC [49].

#### Effect of hydration on the SC proteins

The broad and featureless CP spectrum observed at dry conditions in Fig. 3 is typical for dehydrated proteins where the complete absence of water leads to distortions of the protein structure [50]. Some of the distortions are relaxed already at RH = 80% (24 wt% water) as indicated by the general sharpening of the CP resonances, e.g., in the protein C_α_ region centered around 57 ppm and the peak at 40.6 ppm from Leu C_β_ and Lys C_ε_. The loss of the sharper features for these resonances when increasing the RH from 90% (37 wt% water) and above is tentatively attributed to the onset of keratin core dynamics on the millisecond time scale. The resonance lines are broadened when the protein segments explore the range of available conformations, and consequently chemical shifts, on the (5 kHz)^−1^ time scale of magic-angle spinning [51].

INEPT and DP peaks assigned to Gly C_α_, Ser C_α_, and Ser C_β_ appear at RH = 85% (30 wt% water) and increase upon further hydration. Since these residues are enriched in the terminal domains of the individual proteins that are assembled into keratin filaments, it is reasonable that they are particularly affected by hydration. In analogy with the reasoning for the fluid lipids, the absence of a CP peak with the same shift and line shape as the INEPT signal shows that the terminal domains undergo nearly isotropic reorientation, in agreement with previous solid-state NMR results on isolated and fully hydrated epidermal keratin intermediate filaments [52]. When the rate of molecular reorientation is gradually increasing from the intermediate to the fast dynamical regime, the appearance of the INEPT signal is expected to be preceded by CP and DP signals in close succession even for isotropic motion [14]. Such peaks are not observed in the RH = 80% data, thus indicating that there is an abrupt reduction of *τ*_c_ for the keratin terminals when changing the RH from 80% to 85%. The main increase of the Ser C_β_ DP signal occurs between 80% and 85% RH, while the corresponding INEPT signal continues to increase at higher hydration. For Gly C_α_ and Ser C_α_, there is some further increase in the DP and a pronounced increase of the INEPT signals in the RH interval between 85% and 93% (30 wt% and 46 wt% water). These observations imply that hydration of the keratin takes place at RH above 80%, starting with hydrophilic functional groups such as the hydroxyl of the Ser C_β_ residues at RH = 85%, while further hydration at RH between 85% and 93% leads to free reorientation of the polypeptide backbone of the keratin filament terminal domains. Based solely on CP data, Jokura et al. [53] suggested that the molecular mobility of the keratin filaments increases from the dry state to 30 wt% water content and remains virtually unaffected between 30 wt% and 60 wt% water content. As shown in this work, combining the data from the DP, CP, and INEPT experiments yields a more detailed picture of the events taking place during hydration.

The plastic properties of the SC are important for tolerance to physical stress and it is well known that SC can become non-elastic and brittle in the dry state [54]. The non-elasticity of dry SC can be related to the observed transition from high rigidity to increased mobility of the keratin filaments in the RH interval between 80% and 85%, which also coincides with the onset of swelling of intact SC and isolated corneocytes as observed by sorption microcalorimetry [9] and from diffraction studies on the soft keratin in intact SC [23], [55]. From microscopy studies of SC, it is known that the corneocytes can swell substantially upon hydration, mainly in the vertical dimension [56], [57]. The results in Fig. 3 provide a more detailed molecular picture of this process and suggest that the keratin filaments are densely packed and characterized by rigidity of both the coiled-coil core and the terminal domains of the keratin filaments at dry conditions. Throughout the range of water contents, the core of the keratin filaments remains virtually rigid, while there is a dramatic increase in the mobility of the terminal domains of the keratin filaments upon hydration. The increased mobility of the terminal domains implies that the packing density of the keratin filaments decreases so that the side-chains of the polypeptide backbone in the terminal domains are free to explore configurations in a nearly isotropic manner.

### Heating Affects the SC Molecular Mobility Differently than Hydration

**Figure 4:**
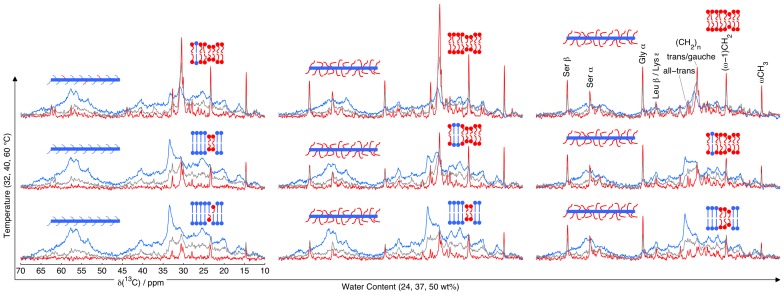
Heating affects the SC molecular mobility differently than hydration. ^13^C MAS NMR spectra of intact SC as a function of temperature and water content using DP (grey), CP (blue), and INEPT (red) pulse sequences. The schematics illustrate the dynamic state of the SC components by color coding according to the observed INEPT (red, mobile) and CP (blue, rigid) intensities of the signature peaks for lipids (all-trans and trans/gauche (CH_2_)_n_, (*ω*−1)CH_2_, *ω*CH_3_), as well as the keratin core (C_α_-region centered around 57 ppm, Leu C_β_, Lys C_ε_) and the protruding terminals of the keratin filaments (Gly C_α_, Ser C_α_, Ser C_β_).

The effect of temperature on SC mobility at different water contents is shown in Fig. 4. For all water contents studied, there is a decrease of the all-trans methylene CP peak at 33.4 ppm and a concomitant increase of the trans/gauche methylene INEPT peak at 30.5 ppm when increasing the temperature from 32 to 60°C. These observations imply a change from a state where the majority of the lipid methylene groups are in a rigid all-trans conformation to a state with fast trans-gauche isomerization and nearly isotropic C-H bond reorientation, thus suggesting a solid-to-liquid phase transition. This transition most likely corresponds to the reversible thermal event of hydrated SC occurring around 55–65°C, as observed by DSC [21], diffraction [8], and IR spectroscopy [21], showing melting of lipids in the same temperature range.

For the SC sample that contains 24 wt% water, there is a small INEPT peak visible at 30.5 ppm at 32°C, which then increases with temperature. This can be compared to the CP signal at 33.4 ppm, which decreases with temperature, although it is still observed at 60°C indicating that a small fraction of solid lipids is still present at this temperature and water content. However, at higher water contents (37 wt% or 50 wt%), the CP peak at 33.4 ppm is absent at 60°C, suggesting that all of the lipids are melted. These observations show that the phase transition occurs at a progressively lower temperature when increasing the water content, in agreement with previous observations from DSC measurements on SC [21], and also analogous to what is observed in other lipid systems [58].

The relative intensities of the mobile lipid INEPT peaks at 14.6, 23.3, and 30.5 ppm are changing with increasing temperature, an effect that is most easily seen in the data for 50 wt% water. The relative increase of the trans/gauche (CH_2_)*_n_* peak at 30.5 ppm results from the melting of progressively longer-chain lipids and, more importantly, from faster dynamics of the fluid lipids upon heating. At 50 wt% water and 32 °C, the hydrocarbon chain CH_2_ segments near the polar headgroup probably have *τ*_c_ above tens of nanoseconds, leading to a reduced intensity of the 30.5 ppm INEPT peak as explained in the previous section. When increasing the temperature, the reduction of *τ*_c_ results in an improved INEPT efficiency for all CH_2_ groups with *τ*_c_ in the range 1–10 ns. The enhancement of the INEPT signal is most pronounced for the fluid lipid segments with the slowest dynamics, i.e. the ones contributing to the 30.5 ppm peak. The observation that the INEPT signal from the (CH_2_)*_n_* resonance is more prominent at 37 wt% water and 60 °C than at 50 wt% water and 60 °C also supports the interpretation that hydration results in a monotonic increase of the fluid lipid fraction, the dynamics of which is gradually slowing down on account of the increasing content of long-chain lipids and cholesterol. In other words, the SC sample at 37 wt% water contains a small fraction of fluid lipids with fast dynamics, while the 50 wt% sample contains a larger fraction of fluid lipids with slower dynamics.

Compared to what was shown for the lipid segments, heating has far less impact on the INEPT intensity of the keratin filaments. At low temperature and hydration, the filaments are rigid, as seen from the dominating broad CP signal in the C_α_ region around 57 ppm and the peak at 40.6 ppm from Leu C_β_ and Lys C_ε_. Increasing the temperature at 24 wt% water leads to the appearance of low-amplitude INEPT peaks at 43.7, 56.7, and 62.4 ppm, indicating the presence of a minor fraction of mobile protruding terminals. For the sample containing 37 wt% water, there is a clear increase of these INEPT signals upon heating, although the effect is minor in comparison to what is observed for the lipids. At 50 wt% water, the increase is barely discernible. A comparison of the INEPT and DP amplitudes at 37 wt% and 50 wt% water shows that the increase of INEPT with temperature is a result of faster dynamics in the transition between the fast-intermediate and fast regimes for polarization transfer. Taken together, the data in Fig. 4 show that both hydration and heating lead to an increasing fraction of mobile SC components, although these events are still different in that they involve different SC components.

### Molecular Insight into the Changes of the SC Permeability Upon Hydration and Heating

A major conclusion from this work is that the increase in RH does not only lead to higher water content in the SC sample, but more importantly, it leads to an increased mobility of the non-aqueous SC components. The observed variations in the physical state of the SC components can provide molecular explanations to previous observations of alterations in SC permeability in response to changes in hydration and temperature. This correlation relies on the definite relation between the physical state of molecular matter and its molecular dynamics (rotational diffusion rate and order parameter) as characterized by PT ssNMR [13], [14], [15]. The SC permeability of a particular compound is primarily attributed to its solubility and diffusional mobility in the different regions of the composite SC membrane, both factors strongly depending on the physical state of the membrane components and their internal organization within the SC membrane [10], [11]. Furthermore, the molecular dynamics of the SC components, and thus their physical state, is not static but can be altered by variations in the external conditions, as shown in this study (Fig. 3 and 4).

Numerous studies have shown that the properties of the SC as a barrier to water and other molecules depend on the external RH [24], [26], [45], and this effect (referred to as skin occlusion) is widely used to increase the permeability of applied drugs. Systematic studies of the diffusional transport of model drugs have demonstrated that the gradient in water activity can be used to regulate skin permeability [24], [59]. The same behavior was predicted from our previous theoretical model describing diffusional transport across responding membranes under steady state conditions for situations where the gradients induce phase transformations within the membrane [11], [12]. The theoretical analysis takes into account that the gradient in water activity across a responding barrier membrane can lead to heterogeneous swelling and phase transformations within the membrane, which in turn affects the molecular environments and thus the local diffusion properties. The detailed analysis was performed for multilayer lipid membranes, as a mimic of SC extracellular lipids [11], and analogous arguments can be applied for hydration-induced phase changes in the protein components. Dehydration leads to a reduction in the water chemical potential, which changes the thermodynamic conditions under which interaggregate interactions occur. This can cause phase changes, as previously shown for lipid systems [58]. For systems that contain charged components, the change in water chemical potential can also alter the local electrostatic interactions and the dissociation equilibrium of the charged components. It is thus possible that the local proton concentration (“pH”) in a multilayer lipid membrane is affected by dehydration [60]. The extracellular SC lipid matrix contain a relatively large fraction of fatty acids, and dehydration may therefore lead to a reduction in the charge density in the lipid headgroup layer, and consequently a closer packing of the hydrocarbon chains in the bilayer [61].

The present data provide qualitative information on the dynamic state of the protein and lipid components in the SC, while there is no direct information about their spatial organization. The data acquired as a function of hydration, shown in Fig. 3, indicate that there is a gradual change in the rate of reorientation of the lipid hydrocarbon chains as a result of changes in the chemical composition of the fluid lipids domains, with increasing fractions of long-chain fatty acids, ceramides, and cholesterol at higher hydration, leading to a slowing down of the dynamics. The observed redistribution of SC lipid species from the solid to the fluid microdomains imply that the latter are located in close proximity of the former, possibly distributed within the main fraction of solid lipids and forming a route for diffusional transport. This interpretation is consistent with previously proposed models of the SC molecular organization, which in general depict the bulk of the lipids as segregated into solid domains with high order connected by microdomains of fluid lipids in a more disordered phase [62], [63], [64].

Previous studies have demonstrated that the SC permeability increases with increasing temperature [25], [27]. The molecular details of how temperature affects the SC lipid lamellae have been characterized by X-ray diffraction and IR spectroscopy methods [7], [8], [21], [22], [28]. From these studies the enhanced permeability of SC can be attributed to thermally induced phase transitions of the lipid matrix [28]. The present results (Fig. 4) are consistent with these observations, and also provide molecular details of the thermal effect on the protein structures, which has not been well characterized until now. As shown in Fig. 4, the keratin filaments are virtually unaffected by temperature at low hydration, while there is a marginal change in the rate of reorientation of the already mobile keratin terminals at higher hydrations.

### Conclusions

The SC membrane separates environments that are profoundly different, and it can be exposed to rather extreme variations in temperature, water, and other compounds that can affect its structure and function. In this work we have performed a detailed ^13^C peak assignment for intact SC, which allowed for an interpretation of the crowded ^13^C NMR spectrum from this multi-component biomaterial. As demonstrated, we are able to capitalize on the peak assignment and map changes in dynamics of different SC molecular components under varying conditions by the use of PT ssNMR. One strong advantage of this method is that it can detect mobile components even when those are occurring in minor fractions, while it simultaneously gives information on the rigid components in the very same sample. Potentially, PT ssNMR can be utilized in other cases of interest, such as common skin disorders like psoriatic and atopic dermatitis, where the biophysical properties are altered resulting in an impaired skin barrier function [65], [66].

The major findings are:

- PT ssNMR provides completely novel information on fluid and solid SC lipids as well as the coiled-coil backbone and protruding terminal domains of the keratin filaments.
- Molecular information on the mobility of cholesterol and lipid carbon chains in ceramides and fatty acids was obtained. The majority of the SC lipids are rigid at 32°C, and those co-exist with a smaller pool of fluid lipids. The fraction of fluid lipids increases with hydration and with temperature.
- In the RH interval between 80% and 85% the keratin filaments undergo a transition between completely solid keratin and a structure with rigid backbone and hydrated mobile terminals. Heating has only a minor influence on the dynamics of the keratin filaments.
- The present data together with previous theoretical treatment and experimental studies of diffusional transport in responding membranes [11], [12], [24] provide a molecular explanation for enhanced permeability of the SC associated with hydration and heating.

## Supporting Information

Figure S1 **^13^C DP MAS NMR spectra of (** ***A*** **) intact SC, (** ***B*** **) isolated corneocytes, (** ***C*** **) SC model lipids, and (** ***D*** **) extracted SC lipids.** Spectra *A* and *B* are scaled to equal intensity at 172.8 ppm, while spectra *A*, *C*, and *D* are scaled to give equal intensity at 30.4–30.6 ppm. The schematics illustrate SC organization of corneocytes, filled with keratin filaments, surrounded by the lipid lamellae matrix. Peaks originating from the keratin and the lipids are assigned in (*B)* and (*C)*. (*E*) Standard numbering of cholesterol carbons and labels of relevant lipid carbons, illustrated here with a ceramide lipid (CER EOS).
(TIF)

Table S1 **^13^C peak assignment of intact pig stratum corneum (SC).**
(PDF)
