# Supplementary figures and images for: Characterization of Stratum Corneum Molecular Dynamics by Natural-Abundance 13C Solid-State NMR
Source: PLoS One. 2013 Apr 23;8(4):e61889. doi: 10.1371/journal.pone.0061889 (PMC3633950; doi:10.1371/journal.pone.0061889)

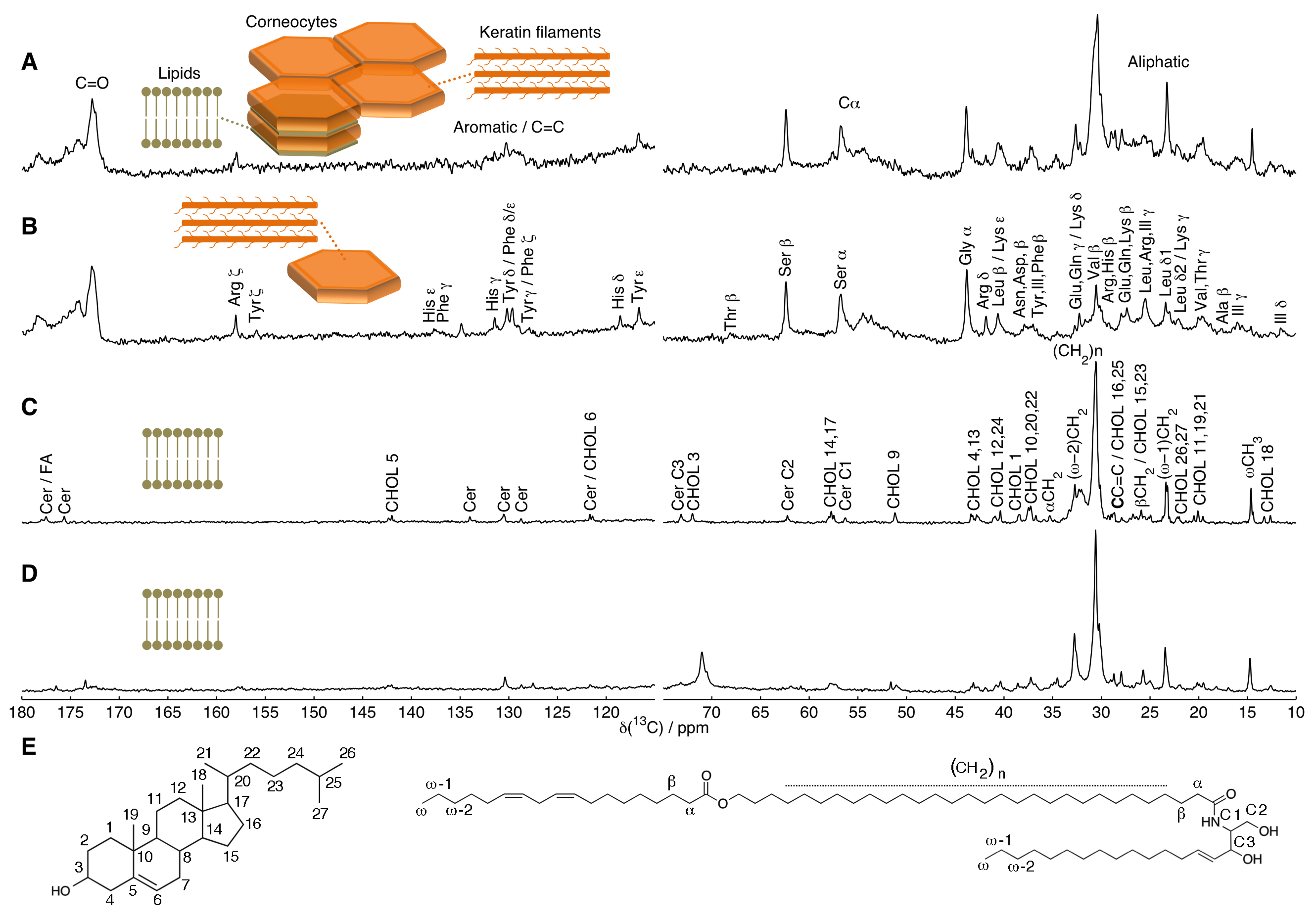

Supplement: Figure S1 — 13C DP MAS NMR spectra of ( A ) intact SC, ( B ) isolated corneocytes, ( C ) SC model lipids, and ( D ) extracted SC lipids. Spectra A and B are scaled to equal intensity at 172.8 ppm, while spectra A, C, and D are scaled to give equal intensity at 30.4–30.6 ppm. The schematics illustrate SC organization of corneocytes, filled with keratin filaments, surrounded by the lipid lamellae matrix. Peaks originating from the keratin and the lipids are assigned in (B) and (C). (E) Standard numbering of cholesterol carbons and labels of relevant lipid carbons, illustrated here with a ceramide lipid (CER EOS). (TIF) [file pone.0061889.s001.tif]
