# Supplementary material for: Characterization of Stratum Corneum Molecular Dynamics by Natural-Abundance 13C Solid-State NMR
Source: PLoS One. 2013 Apr 23;8(4):e61889. doi: 10.1371/journal.pone.0061889 (PMC3633950; doi:10.1371/journal.pone.0061889)
Supplement: Table S1 — 13C peak assignment of intact pig stratum corneum (SC). (PDF) [file pone.0061889.s002.pdf]

TABLE S1. ^{13}C peak assignment of intact pig stratum corneum (SC).

δ (ppm)	Molecule	Segment
11.6	Ill	C_δ
12.7	Cholesterol	C18
13.3	Cholesterol	C18
14.6	Aliphatic	ωCH_3
16.1	Ill	C_γ
17.6	Ala	C_β
19.6	Cholesterol	C21
20.1	Cholesterol	C19
20.5	Cholesterol	C11
19.7-20.1	Val, Thr	C_γ
22.0-22.2	Cholesterol	C26 / C27
22.1	Leu / Lys	$C_{\delta 2}$ / C_γ
23.3	Aliphatic	$(\omega\text{-}1)CH_2$
23.4	Leu	$C_{\delta 1}$
25.0-26.8	Aliphatic, Cholesterol	βCH_2, C15 / C23
25.5	Leu, Arg, Ill	C_γ
27.4-28.0	Glu, Gln, Lys	C_β
27.9-29.0	Aliphatic, Cholesterol	**C**C=C, C25 / C16
29.2	Arg, His	C_β
29.5-31.2	Aliphatic	$(CH_2)_n$
30.5	Val	C_β
32.0-32.7	Aliphatic	$(\omega\text{-}2)CH_2$
32.3	Glu, Gln / Lys	C_γ / C_δ
34.8-36.0	Aliphatic	αCH_2
36.7	Cholesterol	C10
37.0	Tyr, Ill, Phe	C_β
37.3	Cholesterol	C22
37.5	Cholesterol	C20
37.6-37.9	Asn, Asp	C_β
38.4	Cholesterol	C1
40.4	Cholesterol	C24
40.6	Leu / Lys	C_β / C_ε
40.9	Cholesterol	C12
41.8	Arg	C_δ
42.8	Cholesterol	C4
43.4	Cholesterol	C13
43.7	Gly	C_α
≈50-60	All amino acid residues (except Gly)	C_α
51.2	Cholesterol	C9
56.3	Ceramides	C1

Continued

TABLE S1 ***Continued***

**δ (ppm)**	**Molecule**	**Segment**
56.7	Ser	C_{α}
62.3	Ceramides	C2
62.4	Ser	C_{β}
68.1	Thr	C_{β}
72.0	Cholesterol	C3
73.2	Ceramides	C3
116.6	Tyr	C_{ε}
118.6	His	C_{δ}
121.4	Cholesterol	C6
121.7	Ceramides	C=C
127.9	Tyr / Phe	C_{γ} / C_{ζ}
128.7	Ceramides	C=C
129.6	Tyr / Phe	C_{δ} / C_{ε}
130.2	Phe	C_{δ}
130.5	Ceramides	C=C
131.5	His	C_{γ}
134.0	Ceramides	C=C
136.8	Phe	C_{γ}
137.7	His	C_{ε}
142	Cholesterol	C5
155.9	Tyr	C_{ζ}
158.0	Arg	C_{ζ}
172-182	Amino acid residues and SC lipids	C=O
